# Effective *In Vivo* Gene Modification in Mouse Tissue-Resident Peritoneal Macrophages by Intraperitoneal Delivery of Lentiviral Vectors

**DOI:** 10.1016/j.omtm.2020.10.021

**Published:** 2020-11-03

**Authors:** Natacha Ipseiz, Magdalena A. Czubala, Valentina M.T. Bart, Luke C. Davies, Robert H. Jenkins, Paul Brennan, Philip R. Taylor

(Molecular Therapy: Methods & Clinical Development *16*, 21–31; March 13, 2020)

## Main Text

In the originally published version of this article, “sodium thioglycolate (96.5%) (Sigma-Aldrich, cat#T0632)” is listed as a reagent, which is incorrect. It should read “Brewer thioglycolate medium (Sigma-Aldrich, cat#B2551)”.

The authors regret this error.

