# Supplementary material for: Effective In Vivo Gene Modification in Mouse Tissue-Resident Peritoneal Macrophages by Intraperitoneal Delivery of Lentiviral Vectors
Source: Mol Ther Methods Clin Dev. 2019 Oct 18;16:21–31. doi: 10.1016/j.omtm.2019.10.004 (PMC6838965; doi:10.1016/j.omtm.2019.10.004)
Supplement: Document S1. Figures S1–S3 and Tables S1 and S2 [file mmc1.pdf]

OMTM, Volume *16*

Supplemental Information

Effective *In Vivo* Gene Modification in Mouse Tissue-Resident Peritoneal Macrophages by Intraperitoneal Delivery of Lentiviral Vectors

Natacha Ipseiz, Magdalena A. Czubala, Valentina M.T. Bart, Luke C. Davies, Robert H. Jenkins, Paul Brennan, and Philip R. Taylor

Supplementary figure 1

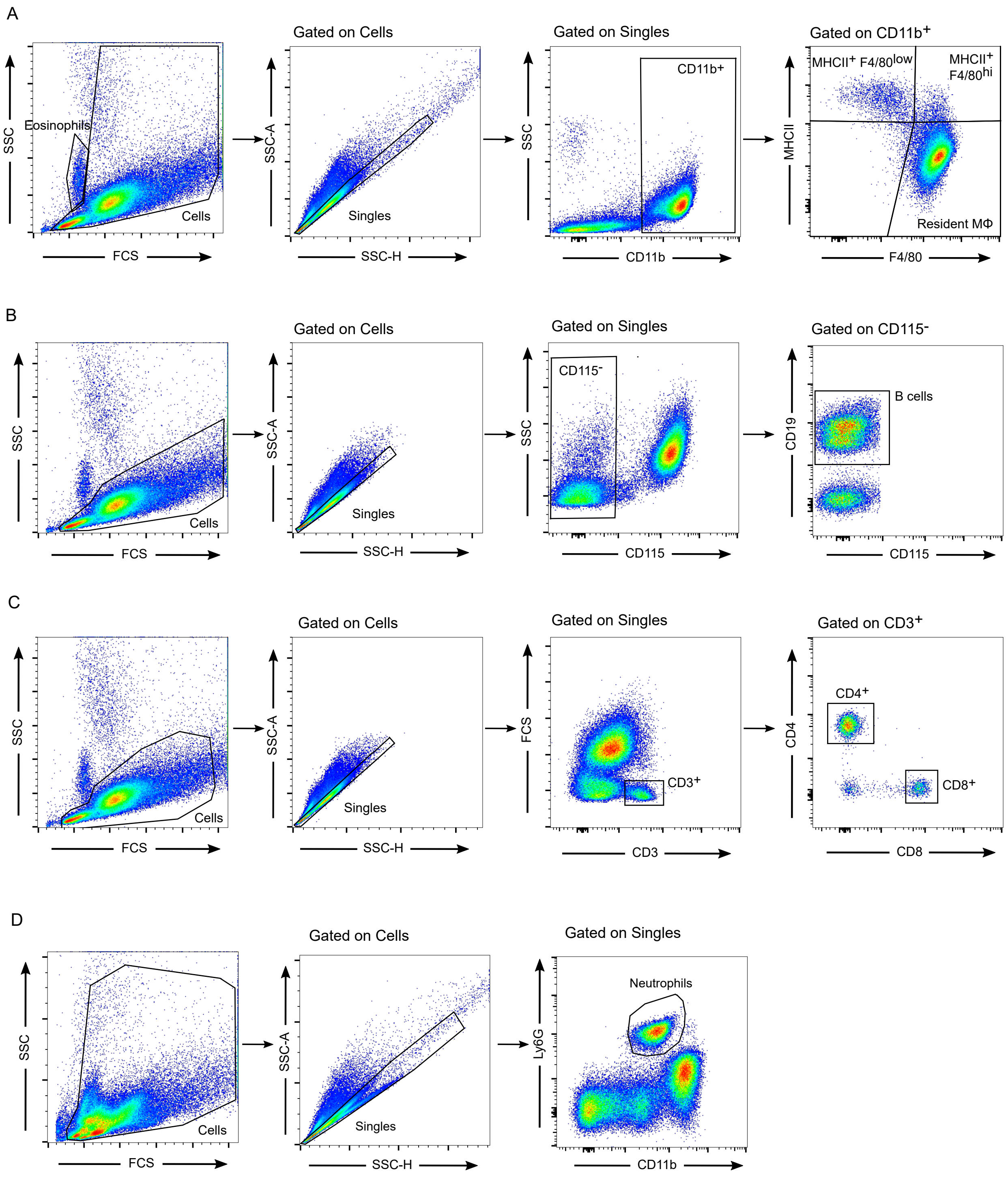

Supplementary figure 2

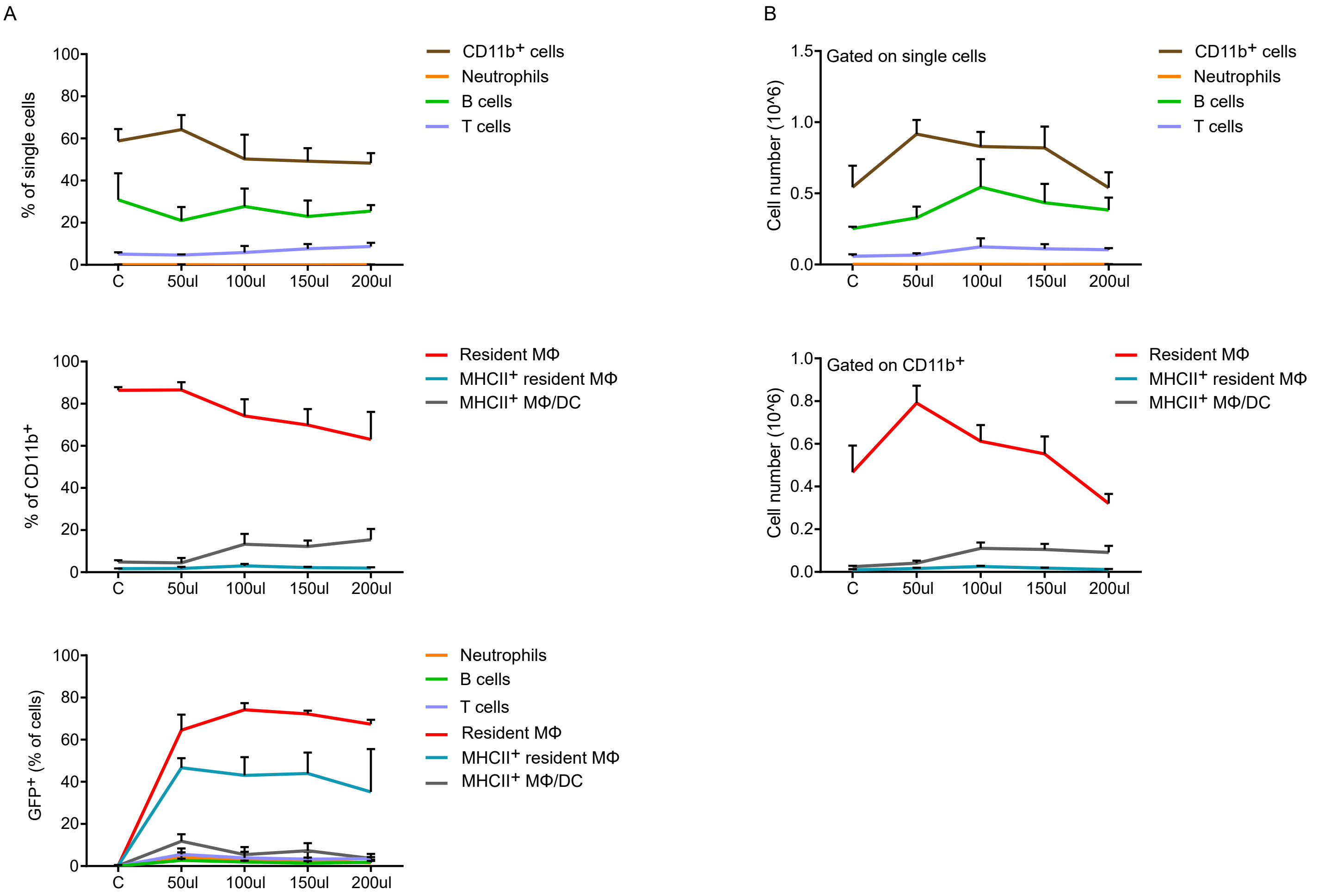

Supplementary figure 3

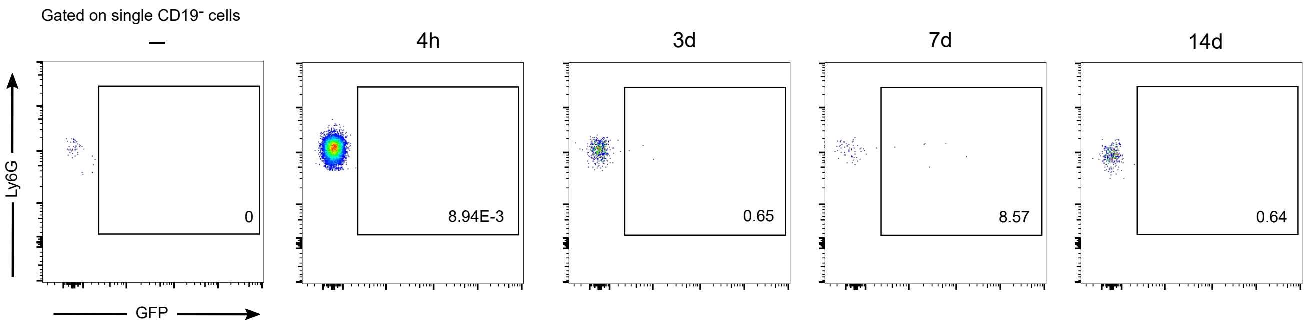

Supplementary Fig.1: Gating strategies used to define the various populations analysed. (A) MΦ/DCs are defined as $CD11b^+$ and $MHCII^+$ $F4/80^-$ cells, after gating on single cells. Eosinophils can be primary identified by their high SSC but would be further specific staining such as SiglecF to be confirmed **(B)** $CD19^+$ B cells are identified after gating on singles cells and $CD115^-$ population. **(C)** $CD4^+$ and $CD8^+$ T cells are found after gating on single cells and $CD3^+$ cells. **(D)** Neutrophils are easily identified as $Ly6G^+$ $CD11b^+$ cells after gating on single cells.

Supplementary Fig.2: Impact of various doses of lentivirus infection on peritoneal inflammation Percentage **(A)** and cell number **(B)** of cells following various amount of *in vivo* lentivirus infection (50, 100, 150 or 200µl in a total volume of 200µl AimV medium), 3 days after i.p. injection.

Supplementary Fig.3: Neutrophils have neglectable GFP expression after lentivirus injection. Flow cytometry analysis of GFP expression in neutrophils after 100ul AimV (-) or lentivirus i.p injection, after the indicated time points.

Supplementary Table 1: Combination of antibodies used to define the various population analysis can be found under Staining 1 to 6. The indicated flow cytometer channels are specific to the Attune flow cytometer with Red, Blue and Violet laser and should only be followed if using the exact same flow cytometer. When using a different one, or an Attune flow cytometer with different lasers, the proper channels should be researched before starting the experiment and the fluorophores used adapted to the available flow cytometer. The same apply to Image Stream channels.

Supplementary Table 2: List of all antibodies used in this protocol.

Staining 1		
Attune channel	Antibodies	Dilution
BL1	GFP	
BL3	MHCII PerCpCy5.5	1/400
BL4	CD11c PeCy7	1/800
RL1	CD226 APC	1/400
RL2	CD11b A700	1/700
VL1	F4/80 Pacific Blue	1/700

Staining 3		
Attune channel	Antibodies	Dilution
BL1	GFP	
BL3	Ly6G PerCpCy5.5	1/400
RL1	CD115 APC	1/400
RL2	CD11b A700	1/700
VL1	CD19 V450	1/400

Staining 2		
Attune channel	Antibodies	Dilution
BL1	GFP	
BL3	CD11b PerCpCy5.5	1/400
RL1	Tim4 A647	1/600
VL1	F4/80 Pacific Blue	1/700

Staining 4		
Attune channel	Antibodies	Dilution
BL1	GFP	
BL3	CD3e PerCpCy5.5	1/200
RL1	CD4 APC	1/400
VL1	CD8a	1/400

Staining 5		
Attune channel	Antibodies	Dilution
BL1	GFP	
BL4	Tim4 PeCy7	1/400
RL2	CD11b A700	1/700
VL1	CD73 ef450	1/400
VL3	F4/80 BV605	1/400

Staining 6		
IS channel	Antibodies	Dilution
Ch2	GFP	
Ch3	HIV-1 Core Antigen	1/100
Ch7	Tim4 A647	1/600
Ch11	F4/80 Pacific Blue	1/700

Table 1

Table 2

Antibody name	Fluorophore	Company	Clone No.	Cat. No.	Dilution used	Final concentration	Stock
HIV-1 Core Antigen	RD1	Beckman Coutler	FH190-1-1	6604667	1/100	1	100 tests
I-A/I-E	PerCpCy5.5	Biolegend	M5/114.15.2	107625	1/400	0.5ug/ml	0.2mg/ml
Ly6G	PerCpCy5.5	Biolegend	1A8	127615	1/400	0.5ug/ml	0.2mg/ml
CD3e	PerCpCy5.5	BD	17A2	560527	1/200	1ug/ml	0.2mg/ml
CD3e	PE/Cy7	Biolegend	500A2	152313	1/400	0.5ug/ml	0.2mg/ml
CD11c	PeCy7	Biolegend	N418	117317	1/800	0.25ug/ml	0.2mg/ml
CD11c	BV605	Biolegend	N418	117333	1/400	0.5ug/ml	0.2mg/ml
CD226	A647	Biolegend	10 E 5	128808	1/400	1.25ug/ml	0.5mg/ml
Tim4	A647	Biolegend	RTM4-54	130007	1/600	0.83ug/ml	0.5mg/ml
CD4	APC	Biolegend	GK1.5	100412	1/400	0.5ug/ml	0.2mg/ml
CD11b	A700	Biolegend	M1/70	101222	1/700	0.71ug/ml	0.5mg/ml
CD11b	PerCpCy5.5	BD	M1/70	550993	1/400	0.5ug/ml	0.2mg/ml
F4/80	Pacific Blue	Biolegend	BM8	123123	1/700	0.71ug/ml	0.5mg/ml
F4/80	BV605	Biolegend	BM8	123133	1/400	0.25ug/ml	0.1mg/ml
F4/80	BV711	Biolegend	BM8	123147	1/400	0.5ug/ml	0.2mg/ml
CD73	ef450	eBioscience	TY/11.8	16-0731-82	1/400	2.5ug/ml	1mg/ml
CD19	V450	BD	1D3	560375	1/400	0.5ug/ml	0.2mg/ml
CD19	APC	Biolegend	1D3	152410	1/400	0.5ug/ml	0.2mg/ml
CD8a	eFluor450	eBioscience	53-6.7	48-0081-82	1/400	0.5ug/ml	0.2mg/ml
SiglecF	BV421	BD	E50-2440	562681	1/400	0.5ug/ml	0.2mg/ml
NK1.1	APC/Cy7	Biolegend	PK136	108724	1/400	0.5ug/ml	0.2mg/ml
FceR1	eFluor450	eBioscience	Mar-1	48-5898-80	1/400	0.5ug/ml	0.2mg/ml
