# Supplementary material for: Effective In Vivo Gene Modification in Mouse Tissue-Resident Peritoneal Macrophages by Intraperitoneal Delivery of Lentiviral Vectors
Source: Mol Ther Methods Clin Dev. 2019 Oct 18;16:21–31. doi: 10.1016/j.omtm.2019.10.004 (PMC6838965; doi:10.1016/j.omtm.2019.10.004)
Supplement: Document S2. Article plus Supplemental Information [file mmc2.pdf]

Molecular Therapy
Methods & Clinical Development
Protocol

Effective *In Vivo* Gene Modification in Mouse Tissue-Resident Peritoneal Macrophages by Intraperitoneal Delivery of Lentiviral Vectors

Natacha Ipseiz,[1,4] Magdalena A. Czubala,[1,4] Valentina M.T. Bart,[1] Luke C. Davies,[1] Robert H. Jenkins,[1] Paul Brennan,[2] and Philip R. Taylor[1,3]

[1]Systems Immunity Research Institute, Cardiff University School of Medicine, Tenovus Building, Heath Park, Cardiff CF14 4XN, UK; [2]Division of Cancer and Genetics, Cardiff University, School of Medicine, Cancer and Genetics Building, Heath Park, Cardiff CF14 4XN, UK; [3]UK Dementia Research Institute at Cardiff, Hadyn Ellis Building, Maindy Road, Cardiff CF24 4HQ, UK

Tissue-resident macrophages exhibit specialized phenotypes dependent on their *in vivo* physiological niche. Investigation of their function often relies upon complex whole mouse transgenic studies. While some appropriate lineage-associated promoters exist, there are no options for tissue-specific targeting of macrophages. We have developed full protocols for *in vivo* productive infection (defined by stable transgene expression) of tissue-resident macrophages with lentiviral vectors, enabling RNA and protein overexpression, including expression of small RNA species such as shRNA, to knock down and modulate gene expression. These approaches allow robust infection of peritoneal tissue-resident macrophages without significant infection of other cell populations. They permit rapid functional study of macrophages in homeostatic and inflammatory settings, such as thioglycolate-induced peritonitis, while maintaining the cells in their physiological context. Here we provide detailed protocols for the whole workflow: viral production, purification, and quality control; safety considerations for administration of the virus to mice; and assessment of *in vivo* transduction efficiency and the low background levels of inflammation induced by the virus. In summary, we present a quick and accessible protocol for the rapid assessment of gene function in peritoneal tissue-resident macrophages *in vivo*.

INTRODUCTION

Tissue-resident macrophages (MΦs) are phagocytic cells actively contributing to the maintenance of homeostasis and immune surveillance and the resolution of inflammation.[1,2] Their origin varies with their home tissue and is controlled by specific transcription factor expression.[3] In recent years, it has become clear that the physiological environment of tissue-resident MΦs is crucial to their function[4–8] and, hence, *in vivo* study is essential for physiological relevance. Important progress has been made to genetically target MΦs *in vivo*.[9] Particularly, the introduction of alternative envelopes[10] and MΦ-specific synthetic promoters[11] have been proposed to alter the precision of MΦ lineage targeting. Here, we employ a commonly used broadly tropic vesicular stomatitis virus envelop glycoprotein (VSV-G) pseudotyped lentivirus vector with transgenes driven by spleen focus-forming virus (SFFV) promoter and show a natural propensity for peritoneal MΦ (pMΦ) infection after intraperitoneal (i.p.) injection. Lentiviral vectors[12] have proven to be excellent tools to modify gene expression in both human and murine cells.[13–17] In this context, we describe an optimized protocol allowing predominant tissue-resident peritoneal MΦ gene expression *in vivo*, resulting in successful genetic modification of peritoneal MΦs in their physiological environment.[18] Being highly flexible, this technique can be used to either overexpress or downregulate gene expression in peritoneal MΦs both at steady state and during inflammation.

MATERIALS

Reagents

- DMEM (1×) + 4.5 g/L D-glucose, 400 μM L-glutamine (Thermo Fisher Scientific)
- RPMI 1640 medium (1×) + 400 μM L-glutamine (Thermo Fisher Scientific)
- AimV medium (Thermo Fisher Scientific)
- Fetal calf serum (FCS) (heat-inactivated for 30 min at 56°C)
- Penicillin/streptomycin (100×, 10,000 U/mL) (Life Technologies, cat#15140122)
- Sterile Dulbecco's PBS (DPBS) (1×) Mg^{++}- and Ca^{2+}-free (Gibco, cat#14190144)
- 0.05% Trypsin-EDTA (1×) (Trypsin 500mg/L or 0.02mM) (Gibco, cat#25300054)
- 20% (w/v) Sucrose
- Sodium hypochlorite (bleach, 2,000 ppm)

Received 16 April 2019; accepted 14 October 2019;
https://doi.org/10.1016/j.omtm.2019.10.004.

[4]These authors contributed equally to this work.

Correspondence: Philip R. Taylor, Systems Immunity Research Institute, Cardiff University School of Medicine, Tenovus Building, Heath Park, Cardiff CF14 4XN, UK.
E-mail: taylorpr@cardiff.ac.uk

This is an open access article under the CC BY license (http://creativecommons.org/licenses/by/4.0/).

- Hydrex surgical scrub, chlorhexidine gluconate 4% w/v skin cleanser (Ecolab, cat#3037170)
- 4% (w/v) Paraformaldehyde (PFA)
- Effectene transfection reagent (QIAGEN, cat#301425)
- LIVE/DEAD fixable near-IR dead cell stain kit (Thermo Fisher Scientific, cat#L34975)
- Sodium thioglycolate (96.5%) (Sigma-Aldrich, cat#T0632)
- Collagenase type IV (Sigma-Aldrich, cat#C5138)
- DNase I (Sigma-Aldrich, cat#11284932001)
- Hyaluronidase (Sigma-Aldrich, cat# H3506)
- Saponin (Sigma-Aldrich, cat# S4521)

Equipment

- CO_2 incubator
- Cat II biology safety cabinet
- Cell culture flask 175cm^2, 550 mL (CellStar, T175 flask)
- Sterile VWR disposable transfer pipets 23.0 mL, 30 cm (VWR)
- Conical centrifuge tubes (15 mL and 50 mL)
- Microcapillary pipettes (volume range 0.5–1,000 μL)
- Syringes (50 mL and 10 mL)
- 0.5 mL U-100 insulin syringe with needle, 0.33 mm, 29G × 12.7 mm (BD MicroFine+)
- Sterile 23G ×1” 0.6 mm × 25 mm nr16 needles (BD Microlance)
- 0.45 μm Sterile millex GP filter (Millipore, cat#SLHP033RS)
- 40 μm Strainer (Thermo Fisher Scientific, cat# 22363547)
- Sterile 24-well cell culture plate
- U-bottom 96-well cell culture plate
- Centrifuge tubes, conical bottom tubes 25 × 89 mm (Beckman Coulter, cat#358126)
- Centrifuges (ultracentrifuge and TC centrifuge)
- 1.5 mL O-ringed screw tubes (or cryotubes)
- Forceps
- Surgical scissors
- Petri dish
- Needle-proof container
- Flow cytometer
- Ultracentrifuge (Beckman Coulter)
- Fluorescent tissue culture microscope, e.g., EVOS FL (Thermo Fisher Scientific)
- Mice (mice described in this protocol were C57BL/6 females, aged 8–12 weeks and obtained from Charles River)

Reagent Setup

Plasmids

The pCMV-ΔR8.91 packaging plasmid encodes Gag-Pol HIV proteins driven by the cytomegalovirus (CMV) promoter.[19] pMD2.G encodes the VSV-G expressing plasmid driven by CMV promoter.[20]

The EGFP expressing vector was pHR’SIN-cPPT-SEW plasmid. EGFP is downstream of the SFFV promoter and upstream of the Woodchuck hepatitis virus enhancer.[18] All plasmids are ampicillin resistant in bacteria.

Cells

HEK293T cells were maintained in DMEM supplemented with 10% FCS, 100 U/mL penicillin, and 10 μg/mL streptomycin (complete DMEM [cDMEM]). Jurkat cells were maintained in RPMI 1640 supplemented with 10% FCS, 100 U/mL penicillin, and 10 μg/mL streptomycin (cRPMI). Both cell lines were kept at 5% CO_2 and 37°C. It is crucial to ensure the low passage and healthy condition of both cell lines for optimal results. Specifically, poor viability of HEK293T cells will result in low lentiviral yield. Both cell lines should be defrosted at least 1 week prior to usage to recover from freezing. Cells should be mycoplasma free.

Mice

All animal work was conducted in accordance with Institutional and UK Home Office guidelines. Users should make sure their own animal licence authorize work with lentivirus in mice.

Flow Cytometry Buffer

Fluorescence-activated cell sorting (FACS) buffer is used during flow cytometry staining, but can also be used for lavage of the peritoneal cavity (as an alternative to 5 mM EDTA in PBS), and contains 4% heat-inactivated and 0.22 μm filtered FCS and 1 mM sterile EDTA (Affymetrix) in sterile PBS. Perm buffer for intracellular staining contains additional 0.5% w/v saponin.

Blocking Buffer

Blocking buffer contains 10% (v/v) rat serum, 4 μg/mL 2.4G2 antibody (made in house), and 1 mM EDTA in sterile PBS. 2.4G2 antibody minimizes nonspecific binding of antibodies by blocking FcγR receptors II and III and thus is a highly recommended component of the blocking buffer for staining Fc receptor expressing cells.

20% Sucrose

Sucrose (20%w/v) was dissolved in Milli-Q water and sterile filtered using 0.22 μM filters. Aliquots can be stored at 4°C.

Thioglycolate

Thioglycolate was obtained from Sigma-Aldrich, and 4% stock solution was prepared in water. The solution was autoclaved and kept frozen prior to use. For the experiment, 0.1 mL of 4% thioglycolate was injected i.p.

Equipment Setup

The safety and good working condition of the ultracentrifuge must be confirmed before use. A detailed description of the ultracentrifuge setup is provided in 2.2. Lentivirus Purification. We recommend decontaminating buckets and lids after each centrifugation with 70% (v/v) ethanol. Avoid using cleaning detergents that can harm anodized aluminum.

PROCEDURE

The protocol below is optimized for transfection of a T175 flask of HEK293T cells, which corresponds to 1 mL (or 1.5 mL if using Option B in 2.1.2 Lentivirus Collection) of final lentivirus preparation. The

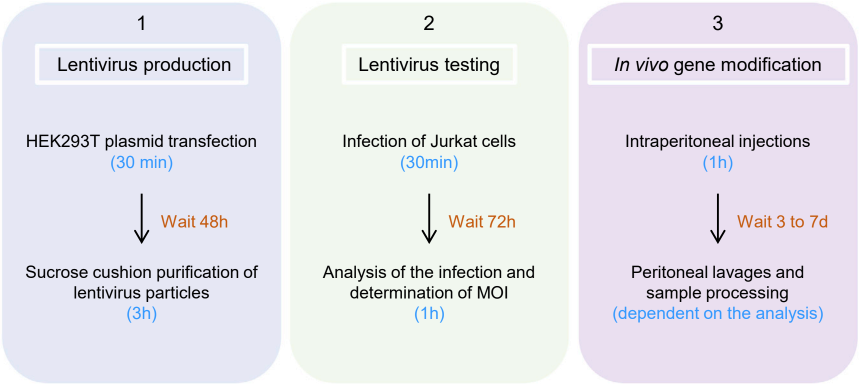

Figure 1. Time Frame Summarizing the Workload from Lentivirus Production to *In Vivo* Testing

The protocol can be divided in 3 major steps: (1) lentivirus production, which takes 2 days; (2) lentivirus *in vitro* testing, which takes 3 days; and (3) *in vivo* gene modification, which can take up to 7 days. Most of the time indicated here is waiting time (orange), and the actual active manipulation time is indicated in blue.

time frame and overview of the complete protocol is shown in Figure 1. Lentivirus-mediated modification of cells is widely used in studies, and this protocol has been optimized for effective infection of mouse tissue-resident peritoneal MΦs. However, pure lentiviral preparation can also be utilized for *in vitro* infections. For the purpose of this paper, we used EGFP as the main readout.

1. HEK293T Cell Transfection (Day 1)

1.1. HEK293T Cell Defrosting and Preparation (Day –6)

HEK293T cells should be stored frozen in liquid nitrogen for long-term storage. We recommend defrosting cells 1 week prior to the start of lentivirus production. It is essential that cells are healthy and in good numbers to achieve the best results from the below protocol. HEK293T cells require a passage every 2–3 days if grown in optimal conditions (up to 70%–80% confluency). Ensure your cells are healthy before starting the protocol.

1.2. HEK293T Cell Seeding for Transfection (Day 0)

One day before transfection, adherent HEK293T cells are freshly seeded in appropriate culture volumes. The cells are gently washed with sterile room temperature PBS (add PBS to the wall of the flask, avoiding disruption of the cell monolayer). Remove PBS and add 7–10 mL of trypsin (for a T175 flask) to the cells. The cells should fully detach during a 5-min incubation at 37°C in a 5% CO_2 incubator. Inactivate trypsin by adding an equal volume of cDMEM and collect the cells and harvest them by centrifugation for 5 min at 350 *g* at room temperature. Carefully remove the supernatant, resuspend the cells in cDMEM, and count. Seed 10–11 × 10^6 of viable HEK293T cells per T175 flask in 20 mL of cDMEM. Maintain the cells at 37°C in a 5% CO_2 incubator for 1 day before transfection.

1.3. Transfection Using an Effectene Kit (Day 1)

Change the medium of the HEK293T cell monolayer to fresh cDMEM (15 mL), taking care not to disturb the monolayer. Prepare the transfection mix as recommended by the manufacturer by adding lentiviral components: 2 μg lentiviral plasmid encoding your gene of interest, 1.5 μg pCMVΔ8.91, and 1 μg pCMVMD2G. Make up the volume to 600 μL with buffer EC, included in Effectene kit. Add 36 μL of enhancer and mix gently with a 1 mL pipette. We recommend mixing by sucking up part of the liquid and putting it back drop by drop. Repeat several times and incubate at room temperature for 5 min. Add 120 μL of Effectene and mix as above, pipetting up and down approximately 20 times. Incubate at room temperature for 10 min. Top up the volume with 5.2 mL of cDMEM and mix as above, this time using a 5 mL stripette. Using a disposable transfer pipet, collect the whole volume (6 mL) and add drop-wise directly onto the HEK293T cell monolayer. Rock the plate gently side to side to allow equal distribution of the plasmid. Keep the cells for 48 h in a 5% CO_2 incubator at 37°C. Taking caution not to disturb the cell monolayer, check for EGFP (or if needed, the other readout marker used) expression in the transfected cells 24–48 h post transfection. For EGFP detection, we used the EVOS cell imaging system (Thermo Fisher Scientific). Strong EGFP signal can be seen 48 h post HEK293T cell transfection (Figure 2A). EGFP signal intensity can vary depending on the transfer vector content.

1.4. Safety Considerations

Lentivirus is a category II pathogen, and specific safety procedures should be followed when handling infected animals, cells, and tissues (for details, see Box 1). HEK293T cells transfected in this protocol are considered infectious from the point of transfection, and safety precautions should be implemented when handling these cells. Any waste products should be considered as category II contaminated and disposed of according to institutional regulations.

2. Lentivirus Collection and Purification (Day 3)

2.1. Lentivirus Collection

2.1.1. First Collection. 48 h post transfection, some detached HEK293T cells can be observed in the flask. Gently collect the medium from the transfected HEK293T cells without disturbing the cell monolayer and transfer to a 50 mL falcon tube. If you wish to proceed with the *optional second collection* step [see 2.1.2 Second Collection (Optional)], replace the medium with 21 mL of fresh cDMEM without disturbing the cell monolayer and place the flask back in the incubator for an additional 24 h. If not proceeding with the optional second collection step, discard the cells and flask following appropriate category II regulations. Continue with the lentivirus purification (2.2 Lentivirus Purification).

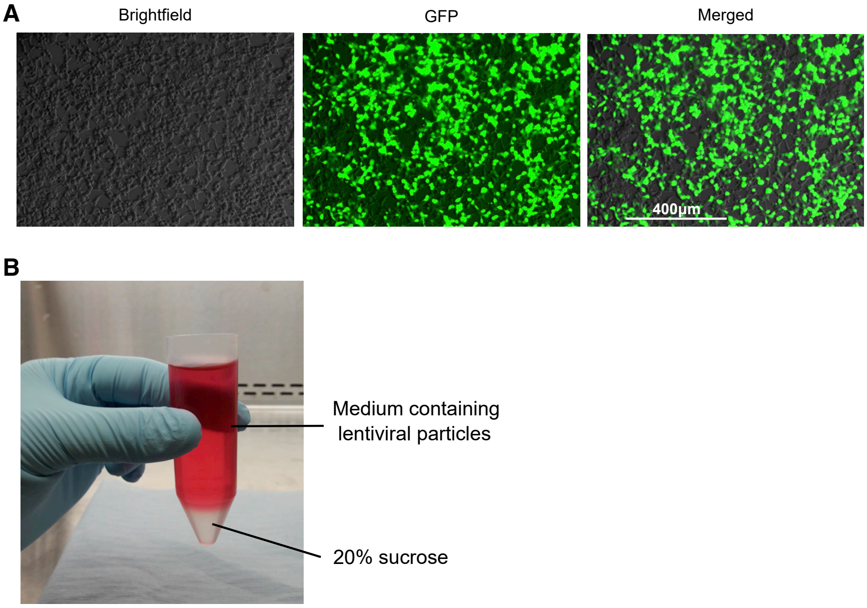

Figure 2. Lentivirus Production from HEK293T Cells and Purification

(A) Representative immunofluorescence pictures of HEK293T cells 48 h after transfection were taken using an EVOS microscope. EGFP is used as a reporter and is inserted in the expression plasmid pSEW. (B) Photograph showing the ultracentrifuge conical tube containing both the layer of 20% sucrose (bottom, clear layer) and medium collected from transfected HEK293T cells (top, red layer).

2.1.2. Second Collection (Optional). 12–24 h post first collection, repeat the above step, but, this time, discard the cells and flask following appropriate category II regulations. Continue with the lentivirus purification (2.2 Lentivirus Purification). It is important to remember that the second lentivirus collection must be titerd separately to the first collection as preparations will have slightly different titer.

2.2. Lentivirus Purification

Pass the collected medium through a 0.45 μm filter into a fresh 50 mL falcon using a 50 mL syringe. It is essential not to use smaller filters (e.g., 0.22 μm) as this will result in the loss of lentivirus particles. Lentivirus particles might bind to cellulose ester membranes and, for better recovery, it is recommended to use low protein-binding polyether sulfone or polyvinylidene fluoride filters.[21] In conical bottom 30 mL ultracentrifuge tubes, first lay 3 mL of 20% sucrose and very carefully overlay the 26 mL of the filtered medium. Two separate layers should be visible (Figure 2B). If the volume of the filtered medium is less than 26 mL, top it up with fresh cDMEM. This will not affect the final lentiviral stock and is essential to avoid collapsing the ultracentrifuge tube during the spin.

We recommend that any staff using the ultracentrifuge receive appropriate training prior to usage to avoid injuries. Once the rotor is securely inserted, close the lid, start the vacuum, and spin the samples at 26,000 rpm for 90 min at 4°C. At the end of the run, carefully remove the rotor and apply class II lentivirus precaution again (see Box 1). Pure lentivirus particles should pellet to the bottom of the tube, but this pellet will not be visible to the naked eye. In one smooth motion, tip out sucrose and medium into a bleach pot and, keeping the tube inverted, place it upside down on paper tissue for 10 min. If any leftover medium accumulates on the tube walls, carefully remove it with a small piece of tissue before turning the tube the right way up. Gently resuspend the lentivirus pellet in 1 mL of AimV medium using a 1,000 μL pipette. When performing the *optional second collection* [see 2.1.2 Second Collection (Optional)], resuspend the lentivirus pellet in 500 μL of AimV medium instead, to ensure comparable titers between the collections. Leave at room temperature for 15 min. Gently mix the virus with the 1,000 μL pipette before aliquoting into 1.5 mL cryotubes. We recommend preparing aliquots of 100 μL, which is the optimal dose for i.p. injection (see 4. *In* Vivo Lentivirus Infection of peritoneal MΦs) plus additional volume to account for pipetting error. We also recommend freezing one vial with about 20 μL of lentivirus stock to be used for titer validation.

3. Lentivirus Infectivity Validation (Days 4–7)

We observed very little variation between lentivirus titers of different preparations when using the same plasmid. However, depending on the plasmid constructs, the infectivity of the lentivirus can change. Therefore, we recommend titration of every lentivirus production and standardization of the injection volume before proceeding with experiments.

An easy and reliable way to estimate virus infectivity is to use healthy Jurkat T cells defrosted 1 week before infection. On the day of the infectivity test, seed Jurkat T cells in 24-well plates at 2×10^5 cells/well in 200 μL of cRPMI1640. On ice, thaw the lentivirus production vial containing 20 μL of the stock (see 2.2. Lentivirus Purification) and mix gently, pipetting up and down. Using serial dilution, infect Jurkat T cells with 0.25, 0.5, 1, 2.5, 5, and 10 μL of lentivirus stock. Keep non-infected Jurkat cells as negative control. Gently rock the plate to assure equal distribution of the virus. Incubate at 37°C for 4 h, and then add cRPMI1640 to reach a total volume of 400 μL per well. 3 days after infection, collect the cells in 1.5 mL collection tubes and centrifuge the cells at 350 *g* at 4°C for 5 min. Discard the supernatant and resuspend the pellet with 2% paraformaldehyde. Leave it for 15 min in the dark. Although EGFP is quite a stable protein, we recommend keeping the samples protected from light from this point on. After fixation, centrifuge the cells at 350 *g* at 4°C for 5 min and resuspend the

Box 1: Recommendation for the Safe Handling of Lentivirus

Lentiviruses are considered class II material and should be handled in accordance with local safety regulations. We recommend each lab to have written standard operating procedures (SOPs) and risk assessment (RA). This is a non-exhaustive list of the precautions we have implemented in our laboratory that may serve as an example, but it needs to be adapted to every local regulation.

- Work under a category II safety hood.
- Double glove when handling contaminated material, and remove the top glove when handling anything not contaminated.
- Special caution should be taken when using sharp objects and their use should be limited to a minimum, with the exception of insulin or single-use safety needles.
- When possible, dedicate a separate incubator for lentivirus.
- Any item coming in contact with contaminated material should be bleached with at least a 2,000 ppm bleach solution for a least 4 h inside the category II hood prior to disposal.
- In case of spillage, immediately cover the contaminated liquid with granulated bleach.

pellet in flow cytometry buffer. Run the cells on a flow cytometer and analyze the percentage of infected cells as well at the mean fluorescent intensity (MFI) of the infected cells, as illustrated in Figures 3A–3C. Jurkat T cells are known to be easily infected by lentivirus,[22] and 5 μL of lentivirus stock should be sufficient to achieve close to 100% infection. Noticeably, the MFI increases with the lentivirus dose (Figure 3C).

It is crucial to test the infectivity of control (empty vector or scrambled short hairpin RNA [shRNA]) and vector of interest (overexpression or targeted shRNA) in Jurkat T cells on the same day to assure accuracy and optimal *in vivo* experiment.

4. *In Vivo* Lentivirus Infection of Tissue Resident peritoneal MΦs (Days 8–22)

All *in vivo* studies with lentivirus should be performed according to local and national guidelines on the ethical use of animals in research as well as adhering to all regulations associated with the use of category II infectious materials. Animal welfare should also be monitored in accordance with local regulations. Injections and collection of samples should follow local authority rules (see Box 1).

Here we describe an optimal working system we have implemented in our laboratory to ensure both the safety of the worker and welfare of the mice. This system may be modified to individual laboratory requirements and local rules.

Prepare lentivirus-containing insulin needles (or single-use needles, 30G for animal welfare and to avoid fluid loss in the needle) within 30 min of planned i.p. injections and store on ice in a lockable box until the time of injection. Drawing the lentivirus up into the syringes should be done in a category II biological safety cabinet considering the safety issues (Box 1). The needle sheath should be placed back on the needle using the one-hand scoop technique. Guide the needle into the sheath using only one hand to hold the syringe and press the sheath against a solid structure such as the inside wall of the safety cabinet or the side of a tip box.

Set up category II biological safety processes prior to injections: our typical setup is shown in Figure 4. Before injections, loosen the sheath of the insulin needle containing the 200 μL lentivirus solution. Manually restrain the mouse with abdomen facing up and head pointed slightly down. Intraperitoneally inject the lentivirus into lower left quadrant of the abdominal cavity, avoiding injection into any peritoneal cavity organs. Still holding the mouse, fill the syringe with 2,000 ppm bleach and safely dispose of it in the sharp safe box. Wipe the injection site on the mouse abdomen with tissue soaked in chlorhexidine gluconate-based disinfectant (such as Hydrex surgical scrub) before returning the animal to the cage. Keep the lentivirus-injected mice in category II scantainers (isolated cages with high efficiency air filtration) for a minimum of 72 h after injection before returning them to category I holding cages and monitor daily. We have never recorded any wellbeing issues caused by lentivirus injection, but extra precaution should be taken when testing this protocol for the first time.

5. Peritoneal Cell Collection from Infected Mice (Days 8–22)

Mice and associated tissue samples are considered category II infectious until up to 72 h after injection. If peritoneal lavage is performed during that time frame, sample collection should be carried out following local category II safety guidelines (see Box 1). Samples are fixed with 2% PFA or lysed.

Euthanize mice by increasing the concentration of CO_2 inhalation and confirm death by cervical dislocation or by following the local animal license recommendation. If samples are collected within 72 h post lentivirus injection, the presence of cell-free lentivirus in tissues is likely, and the following steps must be performed in a category II biological safety cabinet in accordance with safety regulations. Immediately spray the mouse abdomen with 70% isopropanol and carefully cut the skin to expose the peritoneal cavity membrane. Lavage the peritoneal cavity by briskly injecting 6 mL of ice-cold FACS buffer using a 10 mL syringe and 23G needle. Gently roll the mouse to allow the FACS buffer to wash over the peritoneal cavity. Collect the peritoneal fluid using the same syringe and needle, but remove the needle before transferring the cells to 15 mL falcon

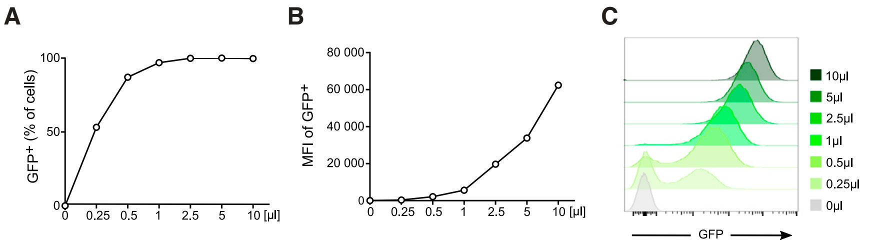

Figure 3. Lentivirus Titer Validation

Representative flow cytometry analysis of Jurkat T cells 72 h after infection with lentivirus containing an EGFP$^+$ plasmid. Percentage of EGFP$^+$ (GFP$^+$) cells (A), mean fluorescence intensity (MFI) of GFP$^+$ cells (B), and histogram representation (C) clearly show the infection rate of the different lentivirus doses.

tubes. To maximize the recovery of the MΦs, a second lavage could be performed as described above. Store the peritoneal lavages on ice until further processing. Dispose of animal carcasses according to local animal guidelines.

6. Peritoneal Cell Staining and Analysis (Day 13)

The viability of the collected cells is expected to be between 98%–100% when measured within 1 h of collection. The number of collected cells can vary depending on mouse strain, treatment, and lavage technique. As a rough indication, about 2.5×10^6 cells usually can be isolated from naive 8–12-week-old C57BL/6 female mice.

Count the collected peritoneal cells and fix them in 2% PFA for 15 min on ice. Wash the cells with cold PBS and distribute them in a V-bottom 96-well plate at 4×10^5 cells per well. Traditional flow cytometric staining protocol can be followed from that step onward. For viability staining (LIVE/DEAD Fixable Near-IR Dead Cell Stain Kit, Thermo Fisher Scientific), the manufacturer instructions were strictly followed (1:1,000 dilution in PBS, staining for 30 min before fixating with 4% PFA for 15 min). For cell surface staining, centrifuge the plate at 350 *g* for 5 min at 4°C and resuspend the pellet in 50 μL blocking buffer. For intracellular staining, prepare the blocking buffer in perm buffer and leave it on ice for 15 min. Add an equal volume of antibody mix prepared in flow cytometry buffer or perm buffer to each well and keep on ice, protected from light, for 30 min. Unstained cells and isotype controls should be included as required. Wash cells twice with ice-cold PBS, centrifuging the plate at 350 *g* for 5 min at 4°C between each wash. Analyze samples on a flow cytometer.

7. Enzymatic Digestion of Organs

For isolation of cells from the spleen, lungs, liver, and lymph nodes, excise and mince the organs with scissors in 1 mL digestion mix containing Hank's balanced salt solution (HBSS) and 2 mg/mL collagenase type IV and 0.03 mg/ml DNase I (additionally supplemented with 1.5 mg/mL of hyaluronidase for lung digestion). Pass the cells through 40-μm strainers and centrifuge for 5 minutes at 350 *g* at 4°C. For lung, spleen, and liver samples, lyse red blood cells using Ammonium-Chloride-Potassium (ACK) lysis buffer (150 mM NH_4Cl, 10 mM $KHCO_3$, 0.1 mM NA_2EDTA, pH 7.2) and pass the samples again through a 40-μm strainer. Continue cell staining and analysis as described in 6. Peritoneal Cell Staining and Analysis (Day 13).

8. Establishment of the Best Dose and Timing for Resident peritoneal MΦ Lentivirus Infection *In Vivo*

Table S1 lists the antibodies and corresponding dilutions used in this protocol to analyze different cell populations in the peritoneal cavity. Gating strategies for peritoneal cavity cell populations can be found in Figure S1.

To establish the optimal lentivirus dose for *in vivo* MΦ studies, we first injected 50, 100, 150, or 200 μL of lentivirus stock in a total volume of 200 μL of AimV medium i.p. and analyzed the infection efficiency 3 days post injection. Injection of 200 μL of AimV medium served as negative control. peritoneal MΦs can be easily distinguished from other cells in the peritoneal cavity by their expression of specific markers: CD11b$^+$ F4/80$^+$ and Tim4$^+$ (Figure 5A). The best infection efficiency of peritoneal MΦs, as measured by EGFP expression, was achieved using 100 μL of lentivirus preparation (Figures 5B and 5C). Injection of higher doses of lentivirus (150 μL and 200 μL) did not improve the percentage of EGFP$^+$ peritoneal MΦs.

Besides using the best dose to obtain the optimal infection efficiency, it is important to consider the best time to harvest the cells. Having identified 100 μL as the optimal dose, we performed a time-dependent experiment where the mice received 100 μL of lentivirus (in a total volume of 200 μL AimV), and peritoneal cells were harvested 4 h, 3 days, 7 days, and 14 days after injection. Based on the longevity of the EGFP signal in infected resident peritoneal MΦs (Figures 5D and 5E), we identified the optimal experimental time being between 3–7 days post i.p. injection. Resident peritoneal MΦ infection can be additionally confirmed by staining the cells for HIV type 1 (HIV-1) core antigen, composed of the 55, 39, 33, and 24 kDa proteins (precursor, intermediate products, and core protein) and analyzing the cells using ImageStream (Figure 5F). The gating strategy of F4/80$^+$ peritoneal MΦs (excluding doublets and cells not in focus) is shown. Clear populations of productively (EGFP$^+$ F4/80$^+$ core antigen$^+$) and unproductively (EGFP$^-$ F4/80$^+$ core antigen$^+$) infected peritoneal MΦs can be distinguished. Unproductive infection results from a failure in lentivirus post cell-entry life-cycle steps, such as reverse transcription or integration, and should be anticipated in cells, especially in MΦs and dendritic cells (DCs) that express lentivirus restriction factors.[23] All peritoneal cells exhibited high viability (single cells), similar to control injected animals (Figure 6A).

It is interesting to note that resident peritoneal MΦs can be further divided into additional subpopulations, such as CD73$^+$ and CD73$^-$

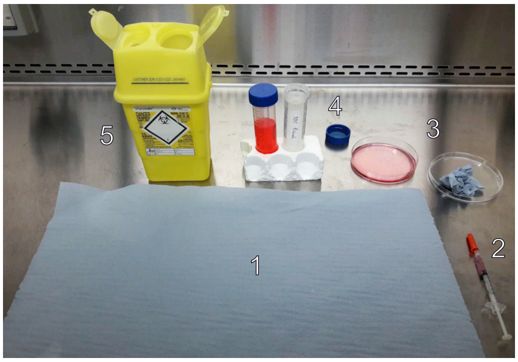

Figure 4. Optimal Setup of the Cat II Hood and Material before *In Vivo* Injection

Photograph illustrating the optimal organization of the workplace, for a right-handed manipulator, before starting *in vivo* work. A left-handed manipulator will have to invert the disposition of all items: (1) clean tissue; (2) insulin syringe loaded with 200 μL AimV medium containing lentivirus and a loose cap; (3) Petri dish containing small pieces of tissue (right) and Hibiscrub (left) to disinfect the mouse after i.p. injection; (4) 50 mL falcon containing 2,000 ppm bleach (right) to bleach the syringe after injection and remaining Hibiscrub (left); (5) sharp safe box to dispose of the insulin syringe after injection.

$Tim4^+$ peritoneal MΦs (Figure 6B), and that these populations appear to be differentially infected by lentivirus (Figure 6C). CD73 is given here as an example, and investigators interested in a particular resident peritoneal MΦ subpopulation should perform similar experiments with the marker of their interest to validate the infection efficiency of their cells of interest. Importantly, we demonstrate that productive infection with lentivirus is limited to resident peritoneal MΦs in the peritoneal cavity, as evidenced by a lack of significant EGFP expression in MΦs in mesenteric lymph nodes (mLNs), lung, livers and spleen 7 days post lentivirus peritoneal challenge (Figure 6D).

We next examined the effectiveness of this protocol under the inflammatory condition (Figures 6E and 6F). Inflammation was induced by i.p. injection of 0.1 mL of 4% thioglycolate, a dose associated with an influx of inflammatory monocyte-derived recruited MΦs coexisting with recoverable resident peritoneal MΦs. We demonstrated that monocytic-like cells ($Ly6C^{hi}$ populations 1 and 2) exhibited minimal evidence of infection. Additionally, our data show that the lentivirus transduction susceptibility of resident MΦs and monocytes (groups 3–5) corresponded to their phenotypic convergence on the recognized phenotype of resident peritoneal MΦs, which remained most easily infected. Monocytic-like cells have previously been reported to be refractory to productive lentivirus infection.[24]

One concern when using i.p lentivirus infection is the potential for lentivirus-induced inflammation. Type I interferons (IFNs) are produced and released in response to viral infections. Therefore, we investigated the IFN response in the mouse peritoneal cavity after lentivirus injection using ProcartaPlex mouse IFN-a/IFN-b 2-plex multiplex immunoassay (Invitrogen) according to the manufacturer's instructions. Both IFNs remained below a detectable level in all samples tested at 4 h and 3, 7, and 14 days post injection, thus indicating a lack or very low level of this cytokine release during lentivirus challenge in the peritoneal cavity (data not shown) in line with previous findings.[25] Thorough analysis of most of the peritoneal cell populations after injection of 100 μL lentivirus (in 200 μL total AimV volume) (Figure 7) showed that only mild inflammation occurs, mainly 4 h after injection, as indicated by the transient influx of a relatively low number of neutrophils (Figures 7A, top graph, and S2). However, these neutrophils did not seem to be productively infected, based on EGFP expression analysis (Figures 7B and S3). B and T cell ($CD19^+$ and $CD3^+$, respectively) percentages and absolute count do not vary over time or with increased doses (Figure S2, top graphs) and they are not infected, as determined by a lack of EGFP positivity. Mast cells, natural killer (NK) cells, and eosinophils also remained $EGFP^-$ (Figure 7B).

Interestingly, the resident peritoneal MΦ population ($F4/80^+$ $Tim4^+$) is stable until day 7 after injection, but it experiences a major drop in percentage (Figure 7A, middle graph) and number (data not shown) between days 7 and 14. Interestingly, the infected cells persist at day 14 in EGFP transgenic mice, suggesting that the drop in resident peritoneal MΦ numbers is, at least in part, due to immune response to the transgene (unpublished data). In contrast, the major histocompatibility complex (MHC) class II^+ resident peritoneal MΦs (MHC class II^+ $F4/80^+$ $Tim4^+$) are also readily infected (up to 60% at day 3 after injection, Figure 7A, lower graph), while their percentage of $EGFP^+$ cells also completely drops by day 14 (Figure 7A, lower graph). Other bone-marrow derived peritoneal MΦs/DCs (MHC class II^+ MΦs/DCs), defined as $CD11b^+$ $CD11c^+$ MHC class II^+, seem to be only minimally affected by the lentivirus in terms of number and infection over time or with increased doses (Figures 7 and S2). We recommend analyzing the infected resident peritoneal MΦs between days 3 and 7 after injection, when the percentage of infected resident peritoneal MΦs is at its highest and inflammation is reduced. In some cases, such as shRNA knockdown of targets, day 7 is preferred to allow time for clearance of pre-existing protein, an approach we used recently to knock down Map3k8 and Gata6 expression *in vivo*.[18]

In summary, the validation of specificity of lentivirus infection during peritoneal cavity injection demonstrates its explicit targeting to tissue resident peritoneal MΦs. High frequency and intensity of EGFP was recorded in those cells with little background infection in other cell populations. A 100 μL dose of lentivirus ensured the best balance between the infection efficiency of target cells and an inflammatory response. However, the optimal amount of lentivirus for injection is strictly dependent on the quality of lentivirus stock obtained following this protocol. We strongly recommend estimating the infectivity of every lentivirus production in Jurkat T cells and scaling the injection volume accordingly.

Timing

HEK293T Cell Transfection (3 Days)

- Day −7: Thaw and passage HEK293T cells at least two times before seeding for lentivirus production

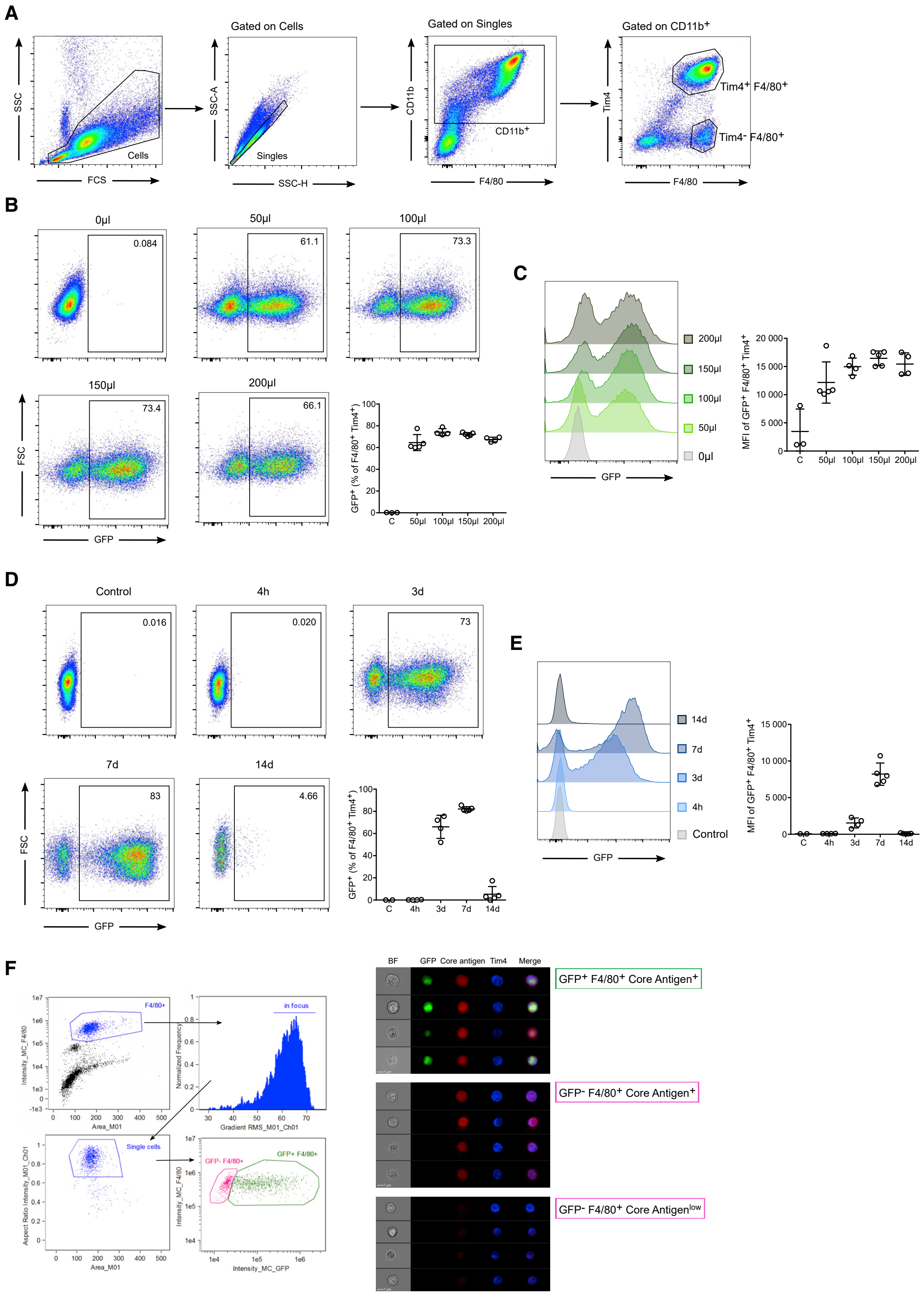

(legend on next page)

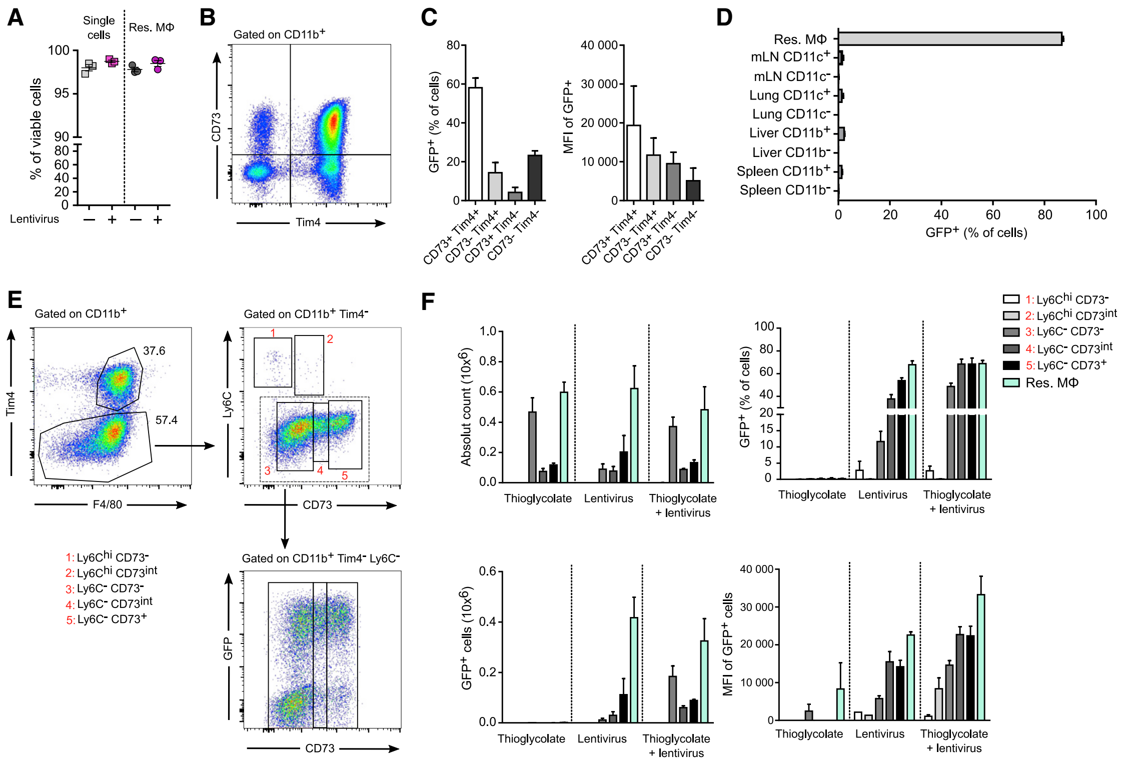

Figure 6. Infection Efficiency of the Resident peritoneal MΦ Subpopulation

(A) Percentage of total single cells and resident MΦs viable 7 days after 100 μL AimV (–) or lentivirus i.p. injection. (B) Gating strategy showing four major populations of peritoneal MΦs that can be found *in vivo*: $CD73^+$ $Tim4^+$, $CD73^-$ $Tim4^+$, $CD73^+$ $Tim4^-$, and $CD73^-$ $Tim4^-$. (C) Percentage of $EGFP^+$ and MFI of $EGFP^+$ of each peritoneal MΦ subpopulation 3 days after infection with 100 μL lentivirus in a total volume of 200 μL AimV medium. (D) Percentage of $EGFP^+$ cells in multiple organs 7 days after lentivirus i.p. injection. mLN, mesenterial lymph node. (E) Gating strategy of peritoneal MΦs and monocytes 3 days after lentivirus i.p. injection. (F) Percentage, MFI, and cell number analysis of $EGFP^+$ monocytes ($Ly6C^{hi}$) and MΦ ($Ly6C^-$). Data are expressed as mean ± SEM.

- Day 0: Count and seed the cells
- Day 1: Transfection
- Day 3: Transfection efficiency check

Lentivirus Collection and Purification (1–2 Days)

- Day 1: Collection, spin, aliquot (if doing the optional second step, add fresh medium to the infected HEK293T cells)
- Day 2 (*Optional*): Second collection

Lentivirus Infectivity (3 Days)

- Day −7: Thaw and passage Jurkat T cells at least two times before infection
- Day 0: Counting and seeding Jurkat T cells and infection
- Day 3: Measurement of infection efficiency by flow cytometry

In Vivo *peritoneal MΦ Infection and Cell Staining (3–7 Days)*

Caution: mice welfare should be monitored daily for the duration of the protocol.

- Day 0: i.p. injection
- Days 3–7: Collection of peritoneal cells and staining and analysis

Troubleshooting

Poor Lentivirus Titer

Poor lentivirus titer could be a result of several issues. Technical issues include low viability of HEK293T cells, high confluency of HEK293T cells on the day of transfection, suboptimal HEK293T cell transfection procedure (e.g., mixing of plasmids, suboptimal plasmid quality, applying transfection reagent to the wall of the flask

Figure 5. Resident Peritoneal Macrophage (peritoneal MΦ) Infection

(A) Gating strategy to define resident peritoneal MΦs. Cells are first gated on singles, followed by $CD11b^+$. Resident peritoneal MΦs are identified as $Tim4^+$ $F4/80^+$ cells. Control non-infected peritoneal lavage was used here, but the strategy is the same for infected lavages. Dot plots (B) and histogram (C) of Flow cytometry analysis of resident peritoneal MΦs isolated 3 days after *in vivo* infection with various amount of lentivirus (50, 100, 150, or 200 μL in a total volume of 200 μL AimV medium). Dot plots (D) and histogram (E) of Flow cytometry analysis of resident peritoneal MΦs isolated 4 h, 3 days, 7 days, and 14 days after *in vivo* infection with 100 μL lentivirus in a total volume of 200 μL AimV medium. (F) Gating strategy and representative pictures of Imagestream analysis of the lentivirus core antigen localization in peritoneal MΦs. Control mice were injected with 200 μL of neat AimV medium. Data are expressed as mean ± SEM.

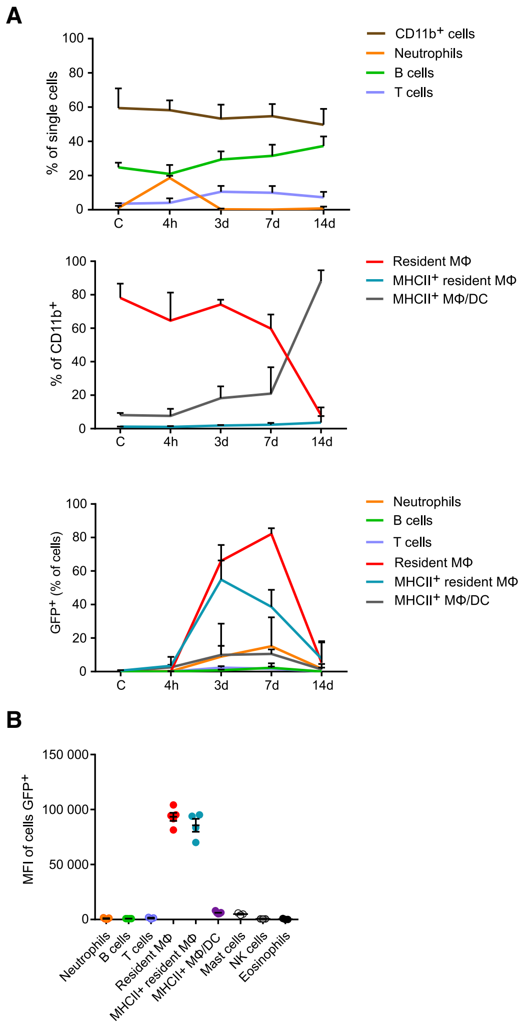

Figure 7. Impact of Lentivirus Infection on Peritoneal Inflammation

(A) Percentage of cells at various times (4 h, 3 days, 7 days, and 14 days) after i.p. injection of 100 μL of lentivirus. (B) MFI of the $EGFP^+$ cells 7 days after the injection of 100 μL of lentivirus. All lentivirus injections were performed with the same total volume of 200 μL (completed by AimV medium). Control mice ("C") received 200 μL of neat AimV medium. Data are expressed as mean ± SEM.

instead of to the cells directly), and usage of a 0.22-μm filter rather than a 0.45-μm filter to filter collected medium. Alternatively, the infectivity of lentivirus preparation can be influenced by the gene inserted in the transfer plasmid. If the gene of interest is very long, it can possibly result in a lower rate of successful reverse transcription and genome integration, resulting in a lower percentage of gene expression.

It is important to note that the permeabilization of cells required when staining for intracellular protein can affect some reporter proteins. We repeatedly observed a strong decrease of EGFP intensity when permeabilizing the cells. In this case, an anti-reporter protein stain should be considered.

High Amount of Lentivirus Stock Required

If a high amount of lentivirus is required for experiments, we recommend preparing an adequate number of flasks for the production (1 × T175 flask = max 1.5 mL of lentivirus preparation) and mixing the final collection from the same time point from all flasks before aliquoting. This ensures reproducible results between the aliquots and reduces the number of samples for titration. When making larger stocks, prepare the plasmids mix (see 1.3 Transfection Using an Effectene Kit) in 50 mL tubes rather than 15 mL to ensure good mixing.

Peritoneal Inflammation after Lentivirus Injection

As we demonstrated, a low level of inflammation can be observed in the peritoneal cavity following lentivirus injection. If major or sustained inflammation is observed, please review your protocol and consider possible contamination when preparing lentivirus.

General Restriction to Further Analyses

Because of the nature of lentiviruses, their use should be reconsidered when further analyses include antiviral response of peritoneal MΦs. VSV-G pseudotyped single round replication HIV-1, similar to the construct employed in this protocol, was shown to induce some of the IFN-stimulated genes (ISGs) in human MΦs in the absence of detectable type-I IFN.[26] Although, we cannot exclude sensing of the lentivirus and low level of antiviral response, our experiments did not detect type I IFNs in the lentivirus-challenged peritoneal cavity.

Lentivirus Storage and Viability

To avoid multiple freeze-thaw cycles, lentivirus preparations should be aliquoted straight after preparation and kept at −80°C. We recommend freezing 100 μL of lentivirus per aliquot covering the optimal injection volume (100 μL) plus possible aliquoting error (20 μL). If injecting more than one mouse, we recommend combining lentivirus aliquots prior to loading of the syringes. We found lentivirus to be viable up to 6 months post preparation if stored correctly. Any longer storage could potentially result in a reduced infection rate, and infectivity of the virus should be tested again.

Safety Concerns when Using Lentivirus

Any procedures covering lentivirus work should be covered in standard operating procedures (SOPs) and risk assessments (RAs) prepared by the laboratory safety officer for each institute in accordance with local and national guidelines. For recommendations regarding safe lentivirus handling, please refer to Box 1.

Anticipated Results

Here, we describe a comprehensive protocol for producing pure lentivirus stocks for *in vivo* peritoneal MΦ gene modification in mice. We explain in detail the lentivirus production method and subsequent steps of lentivirus i.p. injection and data analysis. When followed correctly, the protocol yields a total of 1.5 mL of high-quality lentivirus stock per single preparation. Such volume is equivalent to a minimum of twelve *in vivo* i.p. injections, allowing high-scale studies at reasonable costs. This protocol is, to date, the only one combining rapidity, reproductivity, and high specificity of murine peritoneal MΦ gene targeting.

Although we demonstrated the precise targeting of peritoneal MΦs, low and transient levels of inflammation can be detected in the peritoneal cavity following lentivirus injection, probably due to local response to the viral presence.

This protocol is mostly recommended for short-term investigation (up to 2 weeks after i.p. injection), as the disappearance of peritoneal MΦs 2 weeks post injection was recorded.

SUPPLEMENTAL INFORMATION

Supplemental Information can be found online at https://doi.org/10.1016/j.omtm.2019.10.004.

AUTHOR CONTRIBUTIONS

N.I. and M.A.C. conceived, conducted, and analyzed the experiments and wrote the manuscript. L.C.D. and V.M.T.B, conducted experiments and reviewed the manuscript. R.H.J. and P.B. conceived experiments and reviewed the manuscript. P.R.T. conceived experiments, reviewed the manuscript, and acquired funding.

CONFLICTS OF INTEREST

The authors declare no competing interests.

ACKNOWLEDGMENTS

We would like to thank the staff of our animal facility for the care of the animals during these studies. P.R.T. is funded by a Wellcome Trust Investigator Award (107964/Z/15/Z), and this work was supported in part by the UK Dementia Research Institute. L.C.D. is funded by the Wellcome Trust (103973/Z/14/Z)

REFERENCES

1. Wynn, T.A., Chawla, A., and Pollard, J.W. (2013). Macrophage biology in development, homeostasis and disease. Nature *496*, 445–455.
2. Jantsch, J., Binger, K.J., Müller, D.N., and Titze, J. (2014). Macrophages in homeostatic immune function. Front. Physiol. *5*, 146.
3. Ginhoux, F., and Guilliams, M. (2016). Tissue-Resident Macrophage Ontogeny and Homeostasis. Immunity *44*, 439–449.
4. Amit, I., Winter, D.R., and Jung, S. (2016). The role of the local environment and epigenetics in shaping macrophage identity and their effect on tissue homeostasis. Nat. Immunol. *17*, 18–25.
5. Gosselin, D., Link, V.M., Romanoski, C.E., Fonseca, G.J., Eichenfield, D.Z., Spann, N.J., Stender, J.D., Chun, H.B., Garner, H., Geissmann, F., and Glass, C.K. (2014). Environment drives selection and function of enhancers controlling tissue-specific macrophage identities. Cell *159*, 1327–1340.
6. Lavin, Y., Winter, D., Blecher-Gonen, R., David, E., Keren-Shaul, H., Merad, M., Jung, S., and Amit, I. (2014). Tissue-resident macrophage enhancer landscapes are shaped by the local microenvironment. Cell *159*, 1312–1326.
7. Davies, L.C., and Taylor, P.R. (2015). Tissue-resident macrophages: then and now. Immunology *144*, 541–548.
8. Davies, L.C., Rice, C.M., Palmieri, E.M., Taylor, P.R., Kuhns, D.B., and McVicar, D.W. (2017). Peritoneal tissue-resident macrophages are metabolically poised to engage microbes using tissue-niche fuels. Nat. Commun. *8*, 2074.
9. Shi, J., Hua, L., Harmer, D., Li, P., and Ren, G. (2018). Cre Driver Mice Targeting Macrophages. Methods Mol. Biol. *1784*, 263–275.
10. Markusic, D.M., van Til, N.P., Hiralall, J.K., Elferink, R.P., and Seppen, J. (2009). Reduction of liver macrophage transduction by pseudotyping lentiviral vectors with a fusion envelope from Autographa californica GP64 and Sendai virus F2 domain. BMC Biotechnol. *9*, 85.
11. He, W., Qiang, M., Ma, W., Valente, A.J., Quinones, M.P., Wang, W., Reddick, R.L., Xiao, Q., Ahuja, S.S., Clark, R.A., et al. (2006). Development of a synthetic promoter for macrophage gene therapy. Hum. Gene Ther. *17*, 949–959.
12. Durand, S., and Cimarelli, A. (2011). The inside out of lentiviral vectors. Viruses *3*, 132–159.
13. Kosaka, Y., Kobayashi, N., Fukazawa, T., Totsugawa, T., Maruyama, M., Yong, C., Arata, T., Ikeda, H., Kobayashi, K., Ueda, T., et al. (2004). Lentivirus-based gene delivery in mouse embryonic stem cells. Artif. Organs *28*, 271–277.
14. Escors, D., and Breckpot, K. (2010). Lentiviral vectors in gene therapy: their current status and future potential. Arch. Immunol. Ther. Exp. (Warsz.) *58*, 107–119.
15. Gropp, M., Itsykson, P., Singer, O., Ben-Hur, T., Reinhartz, E., Galun, E., and Reubinoff, B.E. (2003). Stable genetic modification of human embryonic stem cells by lentiviral vectors. Mol. Ther. *7*, 281–287.
16. Hacke, K., Treger, J.A., Bogan, B.T., Schiestl, R.H., and Kasahara, N. (2013). Genetic modification of mouse bone marrow by lentiviral vector-mediated delivery of hypoxanthine-Guanine phosphoribosyltransferase short hairpin RNA confers chemoprotection against 6-thioguanine cytotoxicity. Transplant. Proc. *45*, 2040–2044.
17. Dalsgaard, T., Cecchi, C.R., Askou, A.L., Bak, R.O., Andersen, P.O., Hougaard, D., Jensen, T.G., Dagnæs-Hansen, F., Mikkelsen, J.G., Corydon, T.J., and Aagaard, L. (2018). Improved Lentiviral Gene Delivery to Mouse Liver by Hydrodynamic Vector Injection through Tail Vein. Mol. Ther. Nucleic Acids *12*, 672–683.
18. Rosas, M., Davies, L.C., Giles, P.J., Liao, C.T., Kharfan, B., Stone, T.C., O'Donnell, V.B., Fraser, D.J., Jones, S.A., and Taylor, P.R. (2014). The transcription factor Gata6 links tissue macrophage phenotype and proliferative renewal. Science *344*, 645–648.
19. Zufferey, R., Nagy, D., Mandel, R.J., Naldini, L., and Trono, D. (1997). Multiply attenuated lentiviral vector achieves efficient gene delivery in vivo. Nat. Biotechnol. *15*, 871–875.
20. Naldini, L., Blömer, U., Gallay, P., Ory, D., Mulligan, R., Gage, F.H., Verma, I.M., and Trono, D. (1996). In vivo gene delivery and stable transduction of nondividing cells by a lentiviral vector. Science *272*, 263–267.
21. Mocé-Llivina, L., Jofre, J., and Muniesa, M. (2003). Comparison of polyvinylidene fluoride and polyether sulfone membranes in filtering viral suspensions. J. Virol. Methods *109*, 99–101.
22. Nasri, M., Karimi, A., and Allahbakhshian Farsani, M. (2014). Production, purification and titration of a lentivirus-based vector for gene delivery purposes. Cytotechnology *66*, 1031–1038.
23. Malim, M.H., and Bieniasz, P.D. (2012). HIV Restriction Factors and Mechanisms of Evasion. Cold Spring Harb. Perspect. Med. *2*, a006940.
24. Sonza, S., Maerz, A., Uren, S., Violo, A., Hunter, S., Boyle, W., and Crowe, S. (1995). Susceptibility of human monocytes to HIV type 1 infection in vitro is not dependent on their level of CD4 expression. AIDS Res. Hum. Retroviruses *11*, 769–776.
25. Rasaiyaah, J., Tan, C.P., Fletcher, A.J., Price, A.J., Blondeau, C., Hilditch, L., Jacques, D.A., Selwood, D.L., James, L.C., Noursadeghi, M., and Towers, G.J. (2013). HIV-1 evades innate immune recognition through specific cofactor recruitment. Nature *503*, 402–405.
26. Decalf, J., Desdouits, M., Rodrigues, V., Gobert, F.X., Gentili, M., Marques-Ladeira, S., Chamontin, C., Mougel, M., Cunha de Alencar, B., and Benaroch, P. (2017). Sensing of HIV-1 Entry Triggers a Type I Interferon Response in Human Primary Macrophages. J. Virol. *91*, 91.

OMTM, Volume *16*

Supplemental Information

Effective *In Vivo* Gene Modification in Mouse Tissue-Resident Peritoneal Macrophages by Intraperitoneal Delivery of Lentiviral Vectors

Natacha Ipseiz, Magdalena A. Czubala, Valentina M.T. Bart, Luke C. Davies, Robert H. Jenkins, Paul Brennan, and Philip R. Taylor

Supplementary figure 1

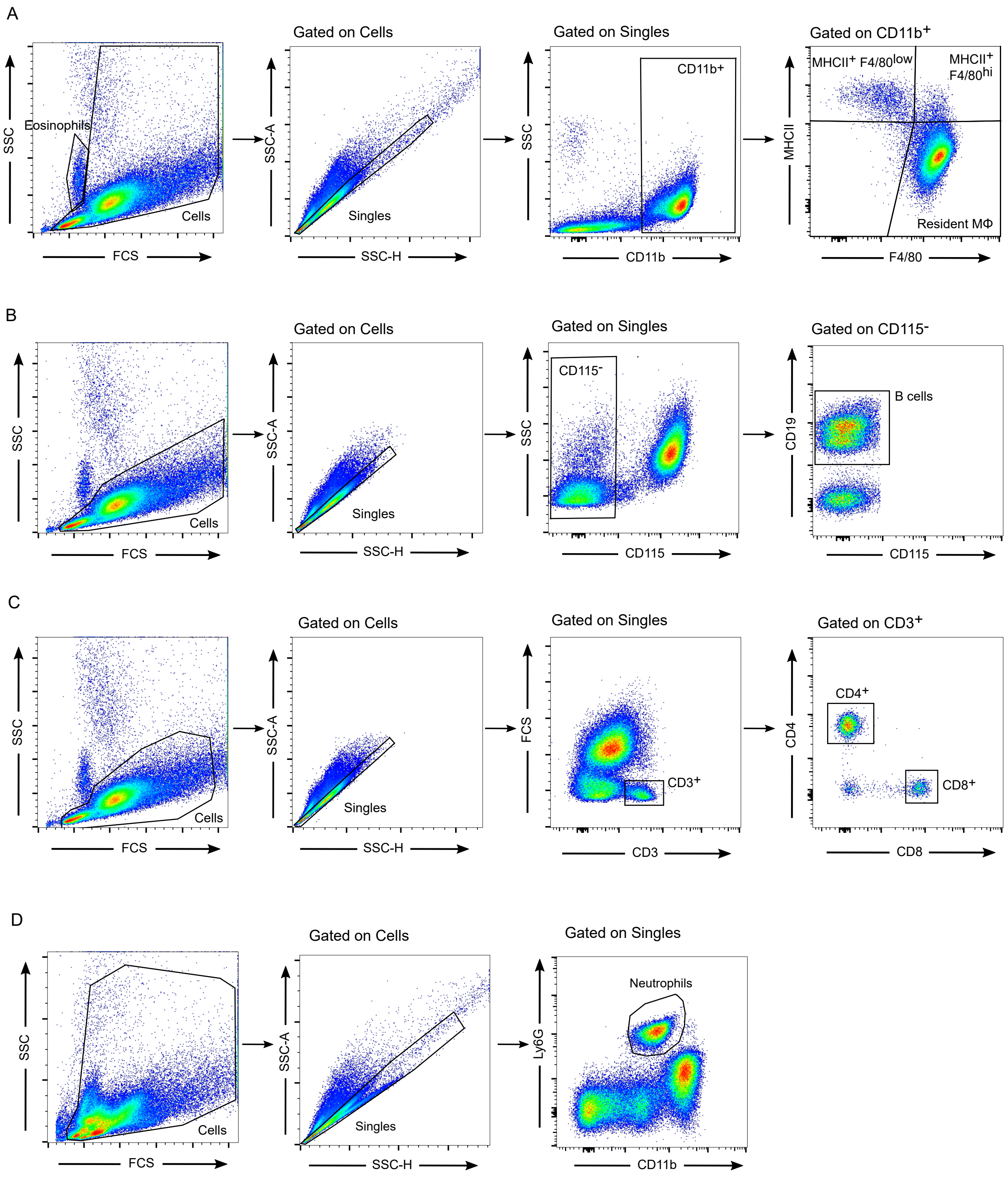

Supplementary figure 2

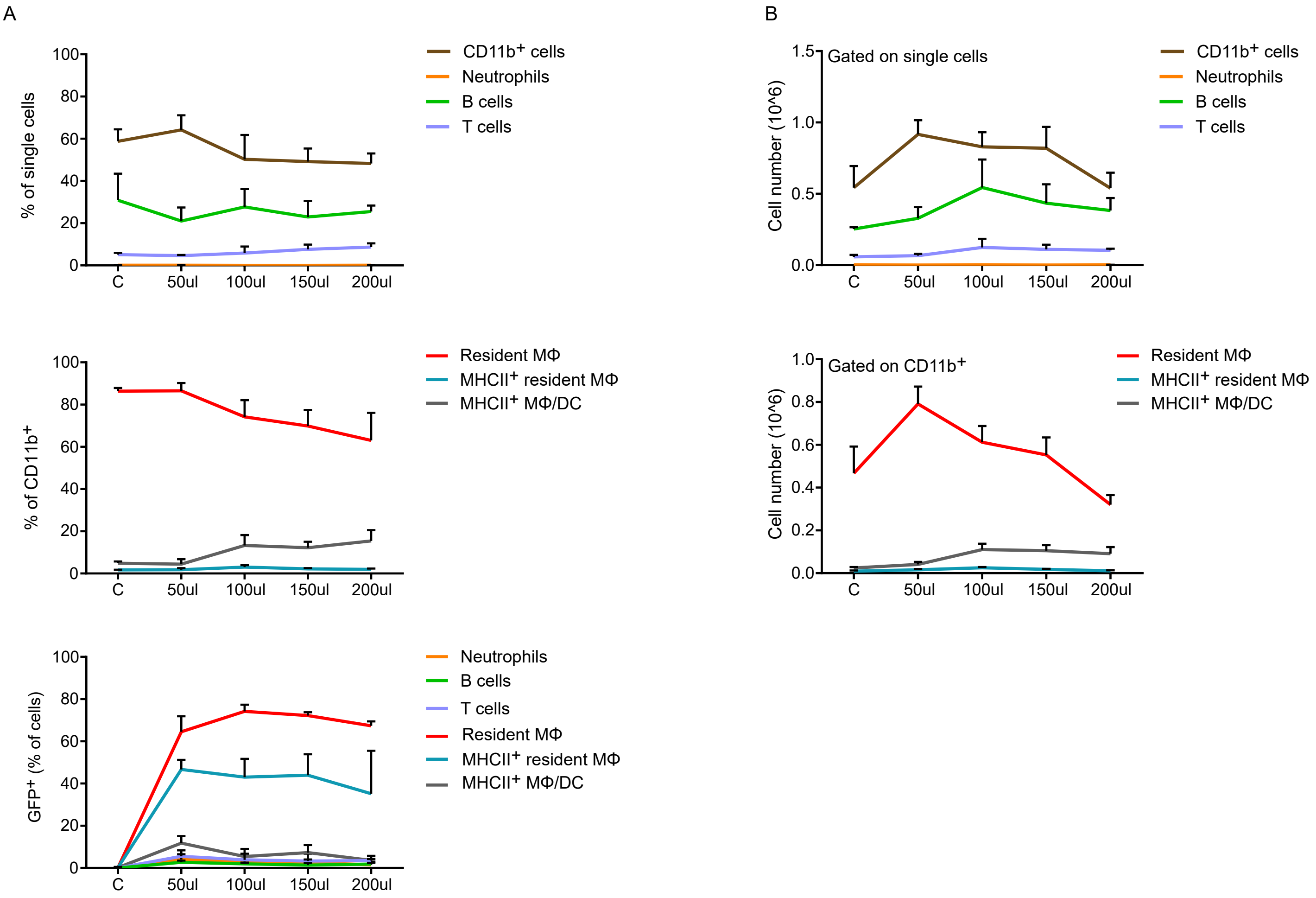

Supplementary figure 3

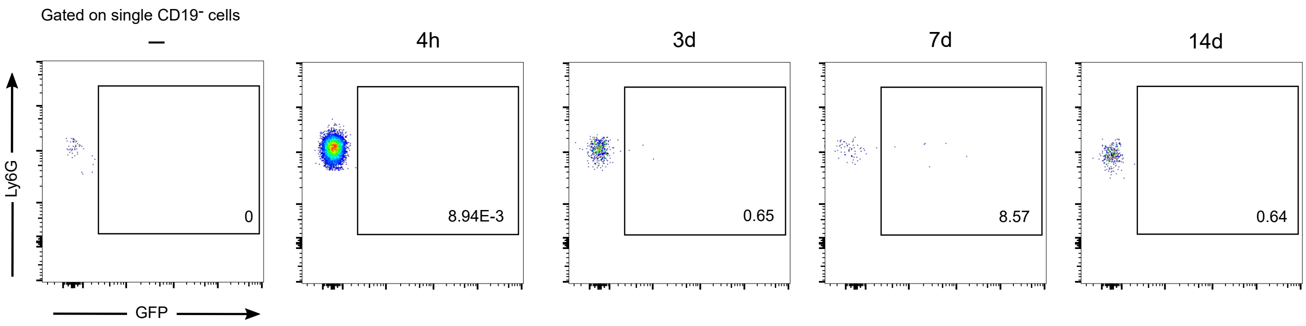

Supplementary Fig.1: Gating strategies used to define the various populations analysed. (A) MΦ/DCs are defined as $CD11b^+$ and $MHCII^+$ $F4/80^-$ cells, after gating on single cells. Eosinophils can be primary identified by their high SSC but would be further specific staining such as SiglecF to be confirmed **(B)** $CD19^+$ B cells are identified after gating on singles cells and $CD115^-$ population. **(C)** $CD4^+$ and $CD8^+$ T cells are found after gating on single cells and $CD3^+$ cells. **(D)** Neutrophils are easily identified as $Ly6G^+$ $CD11b^+$ cells after gating on single cells.

Supplementary Fig.2: Impact of various doses of lentivirus infection on peritoneal inflammation Percentage **(A)** and cell number **(B)** of cells following various amount of *in vivo* lentivirus infection (50, 100, 150 or 200µl in a total volume of 200µl AimV medium), 3 days after i.p. injection.

Supplementary Fig.3: Neutrophils have neglectable GFP expression after lentivirus injection. Flow cytometry analysis of GFP expression in neutrophils after 100ul AimV (-) or lentivirus i.p injection, after the indicated time points.

Supplementary Table 1: Combination of antibodies used to define the various population analysis can be found under Staining 1 to 6. The indicated flow cytometer channels are specific to the Attune flow cytometer with Red, Blue and Violet laser and should only be followed if using the exact same flow cytometer. When using a different one, or an Attune flow cytometer with different lasers, the proper channels should be researched before starting the experiment and the fluorophores used adapted to the available flow cytometer. The same apply to Image Stream channels.

Supplementary Table 2: List of all antibodies used in this protocol.

Staining 1		
Attune channel	Antibodies	Dilution
BL1	GFP	
BL3	MHCII PerCpCy5.5	1/400
BL4	CD11c PeCy7	1/800
RL1	CD226 APC	1/400
RL2	CD11b A700	1/700
VL1	F4/80 Pacific Blue	1/700

Staining 3		
Attune channel	Antibodies	Dilution
BL1	GFP	
BL3	Ly6G PerCpCy5.5	1/400
RL1	CD115 APC	1/400
RL2	CD11b A700	1/700
VL1	CD19 V450	1/400

Staining 2		
Attune channel	Antibodies	Dilution
BL1	GFP	
BL3	CD11b PerCpCy5.5	1/400
RL1	Tim4 A647	1/600
VL1	F4/80 Pacific Blue	1/700

Staining 4		
Attune channel	Antibodies	Dilution
BL1	GFP	
BL3	CD3e PerCpCy5.5	1/200
RL1	CD4 APC	1/400
VL1	CD8a	1/400

Staining 5		
Attune channel	Antibodies	Dilution
BL1	GFP	
BL4	Tim4 PeCy7	1/400
RL2	CD11b A700	1/700
VL1	CD73 ef450	1/400
VL3	F4/80 BV605	1/400

Staining 6		
IS channel	Antibodies	Dilution
Ch2	GFP	
Ch3	HIV-1 Core Antigen	1/100
Ch7	Tim4 A647	1/600
Ch11	F4/80 Pacific Blue	1/700

Table 1

Table 2

Antibody name	Fluorophore	Company	Clone No.	Cat. No.	Dilution used	Final concentration	Stock
HIV-1 Core Antigen	RD1	Beckman Coutler	FH190-1-1	6604667	1/100	1	100 tests
I-A/I-E	PerCpCy5.5	Biolegend	M5/114.15.2	107625	1/400	0.5ug/ml	0.2mg/ml
Ly6G	PerCpCy5.5	Biolegend	1A8	127615	1/400	0.5ug/ml	0.2mg/ml
CD3e	PerCpCy5.5	BD	17A2	560527	1/200	1ug/ml	0.2mg/ml
CD3e	PE/Cy7	Biolegend	500A2	152313	1/400	0.5ug/ml	0.2mg/ml
CD11c	PeCy7	Biolegend	N418	117317	1/800	0.25ug/ml	0.2mg/ml
CD11c	BV605	Biolegend	N418	117333	1/400	0.5ug/ml	0.2mg/ml
CD226	A647	Biolegend	10 E 5	128808	1/400	1.25ug/ml	0.5mg/ml
Tim4	A647	Biolegend	RTM4-54	130007	1/600	0.83ug/ml	0.5mg/ml
CD4	APC	Biolegend	GK1.5	100412	1/400	0.5ug/ml	0.2mg/ml
CD11b	A700	Biolegend	M1/70	101222	1/700	0.71ug/ml	0.5mg/ml
CD11b	PerCpCy5.5	BD	M1/70	550993	1/400	0.5ug/ml	0.2mg/ml
F4/80	Pacific Blue	Biolegend	BM8	123123	1/700	0.71ug/ml	0.5mg/ml
F4/80	BV605	Biolegend	BM8	123133	1/400	0.25ug/ml	0.1mg/ml
F4/80	BV711	Biolegend	BM8	123147	1/400	0.5ug/ml	0.2mg/ml
CD73	ef450	eBioscience	TY/11.8	16-0731-82	1/400	2.5ug/ml	1mg/ml
CD19	V450	BD	1D3	560375	1/400	0.5ug/ml	0.2mg/ml
CD19	APC	Biolegend	1D3	152410	1/400	0.5ug/ml	0.2mg/ml
CD8a	eFluor450	eBioscience	53-6.7	48-0081-82	1/400	0.5ug/ml	0.2mg/ml
SiglecF	BV421	BD	E50-2440	562681	1/400	0.5ug/ml	0.2mg/ml
NK1.1	APC/Cy7	Biolegend	PK136	108724	1/400	0.5ug/ml	0.2mg/ml
FceR1	eFluor450	eBioscience	Mar-1	48-5898-80	1/400	0.5ug/ml	0.2mg/ml
